# New Coleoptera records from New Brunswick, Canada: Trogossitidae, Cleridae, and Melyridae, with an addition to the fauna of Nova Scotia

**DOI:** 10.3897/zookeys.179.2585

**Published:** 2012-04-04

**Authors:** Reginald P. Webster, Jon D. Sweeney, Ian DeMerchant

**Affiliations:** 1 Natural Resources Canada, Canadian Forest Service - Atlantic Forestry Centre, 1350 Regent St., P.O. Box 4000, Fredericton, NB, Canada E3B 5P7

**Keywords:** Cleridae, Melyridae, Trogossitidae, new records, Canada, New Brunswick

## Abstract

*Grynocharis quadrilineata* (Melsheimer) and *Tenebroides corticalis* (Melsheimer) of the family Trogossitidae are newly recorded for New Brunswick, Canada. Additional records of the recently reported *Calitys scabra* (Thunberg)and *Ostoma fraterna* (Randall) are presented for the province. The record of *Ostoma fraterna* is the first recent record of this species from New Brunswick. Additional New Brunswick records of the thaneroclerine, *Zenodosus sanguineus* (Say), are given, indicting that this species is common and widespread in the province. One species of Cleridae, *Cymatodera bicolor* (Say),is newly reported from New Brunswick, and the adventive *Thanasimus formicarius* Linnaeus is newly recorded from Nova Scotia and the Maritime provinces. *Attalus morulus* (LeConte) and *Dolichosoma foveicolle* (Kirby), family Melyridae, are reported for the first time for New Brunswick and the Maritime provinces. Collection, habitat data, and distribution maps are presented for these species.

## Introduction

This paper treats new Coleoptera records from New Brunswick, Canada in the families Cleridae, Melyridae, and Trogossitidae. The fauna of these families from New Brunswick and the Maritime provinces (New Brunswick, Nova Scotia, Prince Edward Island) was recently treated by Majka (2005) (Melyridae), Majka (2006) (Cleridae), and Majka (2011) (Trogossitidae). Intensive collecting in New Brunswick by the first author since 2003 and records more recently obtained from by-catch samples during a study to develop improved lures for the detection of invasive species of Cerambycidae have yielded additional new provincial records in the above families. The purpose of this paper is to report on these new records. In addition, we report a new Nova Scotia and Maritime provinces record for an exotic clerid species. A brief synopsis of each family is included in the results below.

## Methods and conventions

The following records are based on specimens collected during a general survey by the first author to document the Coleoptera fauna of New Brunswick and from by-catch samples obtained during a study to develop a general attractant for Cerambycidae. Additional provincial records were obtained from specimens contained in the collection belonging to Natural Resources Canada, Canadian Forest Service - Atlantic Forestry Centre, Fredericton, New Brunswick.

### Collection methods

Most specimens reported in this paper were collected from Lindgren 12-funnel trap samples during a study to develop a general attractant for the detection of invasive species of Cerambycidae. These traps may visually mimic tree trunks and are often effective for sampling species of Coleoptera that live in microhabitats associated with standing trees (Lindgren 1983). See Webster et al. (in press) for details of the methods used to deploy Lindgren funnel traps and for sample collection. A description of the habitat was recorded for all specimens collected during this survey. Locality and habitat data are presented exactly as on labels for each record. This information, as well as additional collecting notes, is summarized and discussed in collection and habitat data section for each species.

### Distribution

Distribution maps, created using ArcMap and ArcGIS, are presented for each species in New Brunswick, and known distribution in Canada and Alaska is listed for each. New records for New Brunswick are indicated in bold in the Distribution section. The following abbreviations are used in the text:

**Table:** 

**AK**	Alaska	**MB**	Manitoba
**YT**	Yukon Territory	**ON**	Ontario
**NT**	Northwest Territories	**QC**	Quebec
**NU**	Nunavut	**NB**	New Brunswick
**BC**	British Columbia	**PE**	Prince Edward Island
**AB**	Alberta	**NS**	Nova Scotia
**SK**	Saskatchewan	**NF & LB**	Newfoundland and Labrador*

*Newfoundland and Labrador are each treated separately here.

Acronyms of collections examined or where specimens reside referred to in this study are as follows:

**AFC** Atlantic Forestry Centre, Natural Resources Canada, Canadian Forest Service, Fredericton, New Brunswick, Canada

**CNC** Canadian National Collection of Insects, Arachnids and Nematodes, Agriculture and Agri-Food Canada, Ottawa, Ontario, Canada

**NBM** New Brunswick Museum, Saint John, New Brunswick, Canada

**RWC** Reginald P. Webster Collection, Charters Settlement, New Brunswick, Canada

**UMC** Université de Montréal Collection, Montreal, Quebec, Canada

## Results

### Species accounts

All records below are species newly recorded for New Brunswick, Canada, unless noted otherwise. Species followed by ** are newly recorded from the Maritime provinces of Canada.

The classification of the Cleridae follows Optitz (2010); that of the Trogossitidae and Melyridae follows Bouchard et al. (2011).

### Family Trogossitidae Latreille, 1802

Leschen (2002) presented an overview of the North American representatives of the family Trogossitidae (the bark-gnawing beetles). Most species of Trogossitinae are predators and occur under bark or in galleries of wood-boring beetles. A few are minor stored-product pests (Leschen 2002). Larval and adult Peltinae species, such as *Calitys* and *Thymalus marginicollis* Chevrolat, feed on various fruiting bodies of Polyporaceae (Barron 1996). Bousquet (1991) reported two species of Trogossitidae as occurring in New Brunswick: *Ostoma septentrionalis* (Randall) (as *Ostoma columbiana* Casey, see Barron 1996) and the adventive *Tenebroides mauritanicus* (Linnaeus). In a review of the Trogossitidae of Atlantic Canada, Majka (2011) added *Ostoma fraterna* (Randall), *Thymalus marginicollis*, and *Calitys scabra* (Thunberg) to the faunal list of the province. Here, we report two additional species from the province and additional locality data for *Ostoma fraterna*, *Thymalus marginicollis*, and *Calitys scabra* (Table 1).

**Table 1.** Species of Trogossitidae, Cleridae, and Melyridae recorded from New Brunswick, Canada.

**Family Trogossitidae Latreille**
**Subfamily Peltinae Latreille**
**Tribe Lophocaterini Crowson**
*Grynocharis quadrilineata* (Melsheimer)*
**Tribe Peltini Latreille**
*Ostoma fraterna* (Randall)
*Ostoma septentrionalis* (Randall)
**Tribe Thymalini Léveillé**
*Thymalus marginicollis* Chevrolat
**Subfamily Trogossitinae Latreille**
**Tribe Calityini Reitter**
*Calitys scabra* (Thunberg)
**Tribe Trogossitini Latreille**
*Tenebroides corticalis* (Melsheimer)*
*Tenebroides mauritanicus* (Linnaeus)
**Family Cleridae Latreille**
**Subfamily Thaneroclerinae Chapin**
**Tribe Zenodosini Kolibáč**
*Zenodosus sanguineus* (Say)
**Subfamily Tillinae Fischer von Waldheim**
*Cymatodera bicolor* (Say)*
**Subfamily Hydnocerinae Spinola**
*Phyllobaenus humeralis* (Say)
*Phyllobaenus lecontei* (Wolcott)
*Phyllobaenus pallipennis* (Say)
*Phyllobaenus verticalis* (Say)
*Isohydnocera curtipennis* (Newman)
**Subfamily Clerinae Latreille**
*Enoclerus muttkowskii* (Wolcott)
*Enoclerus nigripes nigripes* (Say)
*Enocleris nigripes rufiventris* (Spinola)
*Madoniella dislocata* (Say)
*Thanasimus dubius* (Kirby)
*Thanasimus undatulus* (Say)
*Trichodes nutalli* (Kirby)
**Subfamily Korynetinae Laporte**
*Necrobia violacea* (Linnaeus)
**Family Melyridae Leach**
**Subfamily Malachiinae Flemming**
*Attalus morulus* (LeConte)**
*Collops vittatus* (Say)
*Malachius aeneus* (Linnaeus)
*Nodopus flavilabris* (Say)
**Subfamily Dasytinae Laporte**
*Dolichosoma foveicolle* (Kirby)**

Notes: *New to province, **New to Maritime provinces.

### Subfamily Peltinae Latreille, 1806

**Tribe Lophocaterini Crowson, 1964**

#### Grynocharis quadrilineata

(Melsheimer, 1844)

http://species-id.net/wiki/Grynocharis_quadrilineata

Map 1

##### Material examined.

**New Brunswick, Carleton Co.**, Meduxnekeag Valley Nature Preserve, 46.1907°N, 67.6740°W, 20.VI.2006, R. P. Webster, mature mixed forest, in *Pleurotus* sp. on dead standing *Populus* sp. (1, NBM); same locality and forest type but 7.VI.2007, R. P. Webster, under bark of standing dead beech (4, RWC); Jackson Falls, Bell Forest, 46.2200°N, 67.7231°W, 5-12.VII.2008, R. P. Webster, mature hardwood forest, Lindgren funnel traps (2, AFC, RWC); same locality and habitat data but 23-28.IV.2009, 28.IV-9.V.2009, 14-20.V.2009, 20-26.V.2009, 16-21.VI.2009, R. Webster & M.-A. Giguère, Lindgren funnel traps (7, AFC, RWC). **Queens Co.**, near Queenstown, 45.6904°N, 66.1455°W, 13.V.2008, R. P. Webster, old growth hardwood forest, under bark of standing dead sugar maple (1, RWC); Cranberry Lake P.N.A. (Protected Natural Area), 46.1125°N, 65.6075°W, 24.IV-5.V.2009, 5-12.V.2009, 12-21.V.2009, R. Webster & M.-A. Giguère, old red oak forest, Lindgren funnel traps (4, AFC, RWC); same locality and habitat data but, 21.V.2009, R. Webster & M.-A. Giguère, under bark of red oak (1, AFC); same locality data and forest type, 13-25.V.2011, 25.V-7.VI.2011, M. Roy & V. Webster, Lindgren funnel traps (5, AFC, NBM). **Restigouche Co.**, Jacquet River Gorge P.N.A., 47.804°N, 65.993°W, 13-23.VI.2009, G. J. McBriarty (2, NBM); Dionne Brook P.N.A., 47.9030°N, 68.3503°W, 30.V-15.VI.2011, M. Roy & V. Webster, old-growth northern hardwood forest, Lindgren funnel traps (4, AFC, NBM). **Sunbury Co.**, Acadia Research Forest, 45.9866°N, 66.3841°W, 19-25.V.2009, 2-9.VI.2009, 9-16.VI.2009, 24-30.VI.2009, R. Webster & M.-A. Giguère, mature (110-year-old) red spruce forest with scattered red maple and balsam fir, Lindgren funnel traps (4, AFC). **York Co.**, 15 km W of Tracy off Rt. 645, 45.6848°N, 66.8821°W, 19-25.V.2009, 21-28.VI.2009, R. Webster & M.-A. Giguère, old red pine forest, Lindgren funnel traps (2, AFC); same locality and habitat data, 10-26.V.2010, 4-16.VI.2010, R. Webster & C. MacKay, Lindgren funnel traps (1, AFC, RWC); same locality and habitat data, 8-20.VI.2011, M. Roy & V. Webster, Lindgren funnel trap (1, NBM).

**Map 1.**
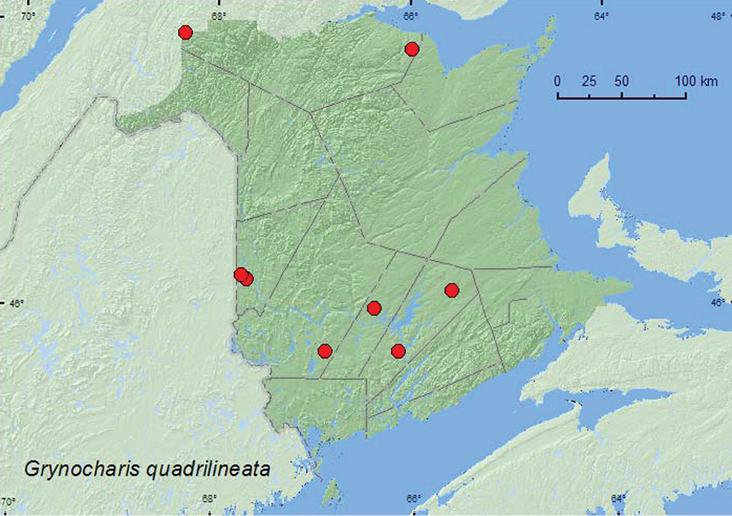
Collection localities in New Brunswick, Canada of *Grynocharis quadrilineata*

##### Collection and habitat data.

In New Brunswick, *Grynocharis quadrilineata*was found in mature and old hardwood forests with sugar maple (*Acer saccharum* Marsh.) and American beech (*Fagus grandifolia* Ehrh.), an old-growth northern hardwood forest with sugar maple and yellow birch (*Betula alleghaniensis* Britt.), an old red oak (*Quercus rubra* L.) forest, a mature (110-year-old) red spruce (*Picea rubens* Sarg.) forest, and an old (180-year-old) red pine (*Pinus resinosa* Ait.) forest. Adults were captured in Lindgren funnel traps in most of these forest types. Specimens with microhabitat data were collected from *Pleurotus* mushrooms on a dead, standing poplar (*Populus* sp.), from under bark of a dead, standing American beech, from under bark of a standing, dead sugar maple, and from under bark of a red oak. This species has been reported from under bark of a dead poplar (Barron 1971). Adults were collected during April, May, June, and July in New Brunswick.

##### Distribution in Canada and Alaska.

MB, ON, QC, **NB,** NS (Bousquet 1991; Majka 2011). This species was first reported from Nova Scotia and the Maritime provinces by Majka (2011) based on one specimen and was considered regionally rare. This species appears to be widespread in New Brunswick and was commonly detected using Lindgren funnel traps.

### Tribe Peltini Latreille, 1806

#### Ostoma fraterna

(Randall, 1838)

http://species-id.net/wiki/Ostoma_fraterna

Map 2

##### Material examined.

**Additional New Brunswick records. Charlotte Co.**, St. Stephen, 17.V.1933, J. B. O’Donnel (8, AFC). **Restigouche Co.**, Dionne Brook P.N.A., 47.9030°N, 68.3503°W, 30.V–15.VI.2011, M. Roy & V. Webster, old-growth northern hardwood forest, Lindgren funnel trap (1, RWC).

**Map 2.**
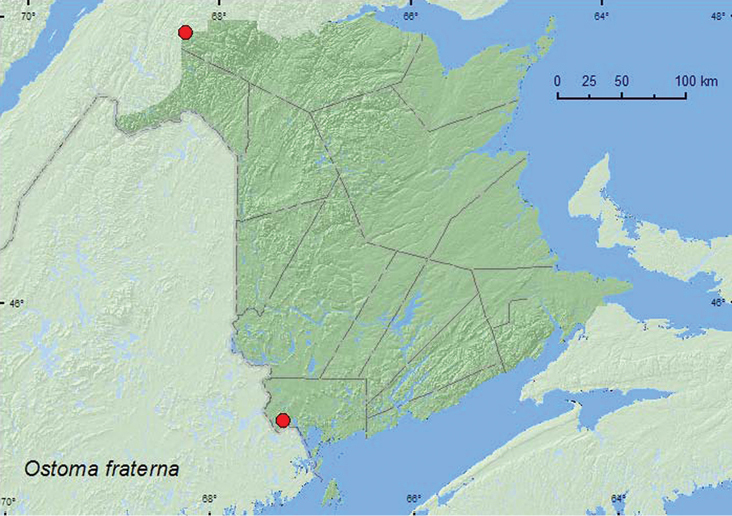
Collection localities in New Brunswick, Canada of *Ostoma fraterna*.

##### Collection and habitat data.

The specimen with habitat data from New Brunswick was collected from a Lindgren funnel trap deployed in an old-growth northern hardwood forest with sugar maple and yellow birch. Specimens were collected during May and June. *Ostoma fraterna* has been found under bark of spruce (*Picea* sp.) and in various Polyporaceae species (*Piptoporus betulinus* (Fr.) Kar., *Spongiporus leucospongia* (Cke. and Hark.) Murr., and *Tyromyces fragilis* (Fr.) Donk) (Barron 1971, 1996).

##### Distribution in Canada and Alaska.

AK, YK, NT, BC, AB, SK, ON, QC, NB, NS, NF (Bousquet 1991; Majka 2011). This uncommon species was first reported for New Brunswick by Majka (2011) based on six specimens collected by W. McIntosh in Saint John during 1902. The record from Dionne Brook Protected Natural Area is the first recent record of this species from the province.

### Tribe Thymalini Léveillé, 1888

#### Thymalus marginicollis

Chevrolat, 1842

http://species-id.net/wiki/Thymalus_marginicollis

Map 3

##### Material examined.

**Additional New Brunswick records. Carleton Co.**, Meduxnekeag Valley Nature Preserve, 46.1907°N, 67.6740°W, 13.VIII.2006, R. P. Webster, mature mixed forest, in polypore fungi (2, RWC); Hartland, Becaguimec Island (in Saint John River), 46.3106°N, 67.5372°W, 13.IX.2006, R. P. Webster, old mixed forest, in large dried polypore fungus (on dead standing basswood) (1, RWC); Jackson Falls, Bell Forest, 46.2200°N, 67.7231°W, 12-19.VI.2008, R. P. Webster, mature hardwood forest, Lindgren funnel trap (1, AFC); same locality and habitat data but 20-26.V.2009, 21-18.VI.2009, 31.VII-7.VIII.2009, R. Webster & M.-A. Giguère, Lindgren funnel traps (4, AFC). **Queens Co.**, Cranberry Lake P.N.A, 46.1125°N, 65.6075°W, 18-25.VI.2009, 21-27.V.2009, 5-11.VI.2009, 11-18.VI.2009, R. Webster & M.-A. Giguère, mature red oak forest, Lindgren funnel traps (5, AFC); same locality data and forest type, 25.V-7.VI.2011, M. Roy & V. Webster, Lindgren funnel trap (1, NBM). **Restigouche, Co.**, Dionne Brook P.N.A., 47.9030°N, 68.3503°W, 30.V-15.VI.2011, M. Roy & V. Webster, old-growth northern hardwood forest, Lindgren funnel traps (3, AFC, NBM); same locality and collectors but 47.9064°N, 68.3441°W, 15-27.VI.2011, old-growth white spruce and balsam fir forest, Lindgren funnel trap (1, NBM). **Sunbury Co.**, Acadia Research Forest, 45.9866°N, 66.3841°W, 2-9.VI.2009, R. Webster & M.-A. Giguère, mature (110-year-old) red spruce forest with scattered red maple and balsam fir, Lindgren funnel traps (2, AFC). **York Co.**, near Browns Mountain Fen, 45.8876°N, 67.6560°W, 3.VIII.2006, R. P. Webster, mature hardwood forest, in slightly dried *Pleurotis* sp. on sugar maple (4, NBM, RWC); Charters Settlement, 45.8286°N, 66.7365°W, 25.VII.2006, R. P. Webster, mixed forest, in polypore fungi on dead (standing) beech (1, RWC); 15 km W of Tracy off Rt. 645, 45.6848°N, 66.8821°W, 1-8.VI.2009, R. Webster & M.-A. Giguère, old red pine forest, Lindgren funnel traps (3, AFC).

**Map 3.**
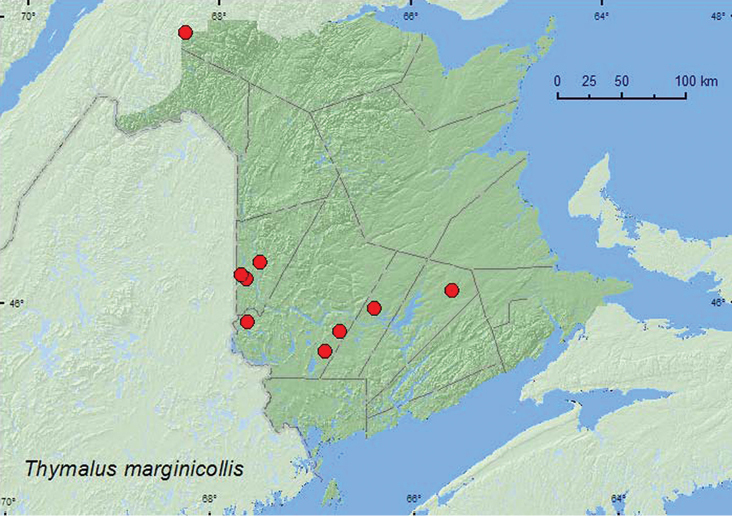
Collection localities in New Brunswick, Canada of *Thymalus marginicollis*.

##### Collection and habitat data.

*Thymalus marginicollis*was collected in Lindgren funnel traps in hardwood forests with sugar maple and American beech, an old-growth northern hardwood forest with sugar maple and yellow birch, an old red oak forest, mixed forests, a mature red spruce forest, and an old red pine forest. Adults were also collected from a large, dried polypore fungus on a dead, standing basswood (*Tilia americana* L.), in a slightly dried *Pleurotus* mushroom on a dead, standing sugar maple, and from a polypore fungi on a dead, standing American beech tree. Barron (1971) reported this species from various polypore species and Majka (2011) reported that it was commonly captured in flight-intercept traps and on the polypore, *Piptoporus betulinus* (Fr.) Kar., growing on white birch (*Betula papyrifera* Marsh.) in Nova Scotia. Adults were captured during May, June, July, August, and September.

##### Distribution in Canada and Alaska.

BC, AB, SK, MB, ON, QC, NB, NS, PE, NF (Bousquet 1991; Majka 2011). *Thymalus marginicollis* was first reported from New Brunswick by Majka (2011) on the basis of one specimen collected by D.F. McAlpine on Todd’s Island (Charlotte Co.) during 2000. This species is common and widespread in New Brunswick and was most commonly detected using Lindgren funnel traps.

### Subfamily Trogossitinae Latreille, 1802

**Tribe Calityini Reitter, 1922**

#### Calitys scabra

(Thunberg, 1784)

http://species-id.net/wiki/Calitys_scabra

Map 4

##### Material examined.

**Additional New Brunswick records. Queens Co.**, Cranberry Lake P.N.A, 46.1125°N, 65.6075°W, 21–27.V.2009, R. Webster & M.-A. Giguère, old red oak forest, Lindgren funnel trap (1, RWC); same locality and habitat data, 25.V–7.VI.2011, 29.VI–7.VII.2011, M. Roy & V. Webster, Lindgren funnel traps (2, NBM, RWC). **Sunbury Co.**, Acadia Research Forest, 11.VI.2008, Brawn/Harrison (2, AFC); same locality but 45.9866°N, 66.3841°W, 13–19.V.2009, 19–25.V.2009, 25.V-2.VI.2009, R. Webster & M.-A. Giguère, mature (110-year-old) red spruce forest with scattered red maple and balsam fir, Lindgren funnel traps (3, AFC, RWC). **York Co.**, 14 km WSW of Tracy, S of Rt. 645, 45.6741°N, 66.8661°W, 10–26.V.2010, R. Webster & C. MacKay, old mixed forest with red and white spruce, red and white pine, balsam fir, eastern white cedar, red maple, and *Populus* sp., Lindgren funnel trap (1, RWC); 15 km W of Tracy off Rt. 645, 45.6848°N, 66.8821°W, 30.V-8.VI.2011, 8–20.VI.2011, M. Roy & V. Webster, old red pine forest, Lindgren funnel traps (6, AFC, NBM, RWC).

**Map 4.**
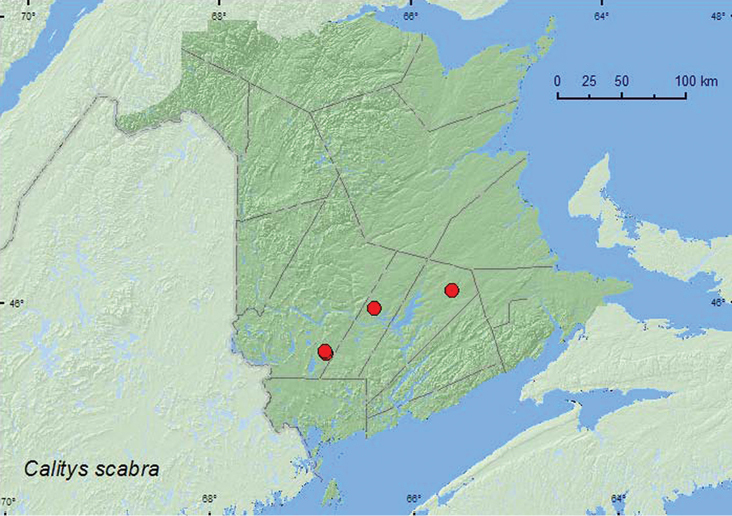
Collection localities in New Brunswick, Canada of *Calitys scabra*.

##### Collection and habitat data.

*Calitys scabra*was captured in Lindgren funnel traps deployed in an old red oak forest, a mature red spruce stand, an old-growth red pine forest, and an old mixed forest. This species was reported from under bark of dead pine and from *Fomitopsis pinicola* (Fr.) Kar. (Barron 1971). Adults were collected during May, June, and July.

##### Distribution in Canada and Alaska.

AK, NT, BC, AB, MB, ON, QC, NB, NS (Bousquet 1991; Majka 2011). Majka (2011) reported this Holarctic species for the first time from New Brunswick based on a specimen collected by W. McIntosh in Saint John, ca. 1900. The above records are the first recent records of this species for New Brunswick.

### Tribe Trogossitini Latreille, 1802

#### Tenebroides corticalis

(Melsheimer, 1844)

http://species-id.net/wiki/Tenebroides_corticalis

Map 5

##### Material examined.

**New Brunswick, Carleton Co.**,Jackson Falls,Bell Forest, 46.2200°N, 67.7231°W, 23-28.IV.2009, 20-26.V.2009, 8-16.VI.2009, R. P. Webster & M.-A. Giguère, mature hardwood forest, Lindgren funnel traps (4, AFC, RWC). **Queens Co.**, Cranberry Lake P.N.A, 46.1125°N, 65.6075°W, 24.IV-5.V.2009, 5-12.V.2009, 12-21.V.2009, 21-27.V.2009, R. Webster & M.-A. Giguère, old red oak forest, Lindgren funnel traps (11, AFC, RWC); same locality data and forest type, 13-25.V.2011, 25.V-7.VI.2011, M. Roy & V. Webster, Lindgren funnel traps (3, NBM). **Sunbury Co.**, Acadia Research Forest, 45.9866°N, 66.3841°W, 16-24.VI.2009, 24-30.VI.2009, 13-21.VII.2009, R. Webster & M.-A. Giguère, mature (110- year-old) red spruce forest with scattered red maple and balsam fir, Lindgren funnel traps (4, AFC). **York Co.**,Charters Settlement, 45.8340°N, 66.7450°W, 2.IV.2005, R. P. Webster, mixed forest, in moss and lichens on tree trunk (1, RWC); same locality and collector but 45.8267°N, 66.7343°W, 16.IV.2005, *Carex* marsh, in litter and sphagnum at base of tree (1, RWC); Nashwaaksis River at Rt. 105, 45.9850°N, 66.6900°W, 6.V.2006, R. P. Webster, river margin in flood debris on upper river margin (1, RWC).

**Map 5.**
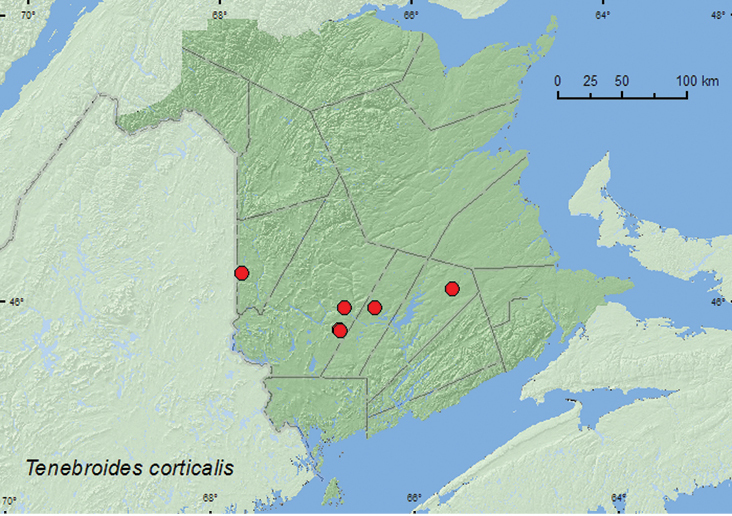
Collection localities in New Brunswick, Canada of *Tenebroides corticalis*.

##### Collection and habitat data.

*Tenebroides corticalis* was captured in Lindgren funnel traps deployed in both deciduous and coniferous forests in New Brunswick. These included a mature hardwood forest with sugar maple, American beech, and white ash (*Fraxinus americana* L.), an old red oak forest, a mixed forest, and a mature red spruce forest. Adults were also collected from moss and lichens on a tree trunk, in litter and sphagnum at the base of a tree in a *Carex* marsh, and in flood debris on the upper margin of a river. Barron (1971) reported this species from under bark of various hardwood species, spruce, and pine. Adults were collected during April, May, June, and July in New Brunswick.

##### Distribution in Canada and Alaska.

AK, YK, NT, BC, SK, MB, ON, QC, **NB,** NS (Bousquet 1991; Majka 2011). Majka (2011) newly recorded this species from the Maritime provinces on the basis of two records from Nova Scotia.

### Family Cleridae Latreille, 1802

The Cleridae (the checkered beetles) prey on other insects as larvae and adults (Opitz 2002). Some species of *Enoclerus* and *Thanasimus* are important control agents of bark beetles and other wood-boring species. Clerids occur under bark, in tunnels of wood and cone borers, on logs and branches, on foliage, and on flowers (Opitz 2002). The Cleridae of the Maritime provinces was reviewed by Majka (2006). Fourteen species were reported for New Brunswick, including *Trichodes nutalli* (Kirby) and *Necrobia violocea* (Linnaeus), which were newly recorded for the province (Majka 2006). Here, *Cymatodera bicolor* (Say)is newly recorded from New Brunswick and *Thanasimus formicarius* Linnaeus is newly reported from Nova Scotia (Table 1).

### Subfamily Thaneroclerinae Chapin, 1924

Kolibáč (1992) separated the Thaneroclerinae from the Cleridae, but this was not followed by Opitz (2002). Later, Optiz (2010) proved conclusively that the Thaneroclerinae is part of the Cleridae. *Zenodosus sanguineus* (Say) is the only member of this subfamily in Canada (McNamara 1991) and the Maritime provinces (Majka 2006).

### Tribe Zenodosini Kolibáč, 1992

#### Zenodosus sanguineus

(Say, 1835)

http://species-id.net/wiki/Zenodosus_sanguineus

Map 6

##### Material examined.

**Additional New Brunswick records. Carleton Co.**,Jackson Falls,Bell Forest, 46.2200°N, 67.7231°W, 13.VIII.2006, R. P. Webster, hardwood forest, in decaying fleshy polypore fungi (1, RWC); same locality data, collector, and forest type, 6.V.2007, in partially dried polypore fungus on dead tree (1, RWC); same locality data, collector, and forest type, 4-12.VI.2008, Lindgren funnel trap (1, AFC); same locality and forest type but 23-28.IV.2009, R. P. Webster & M.-A. Giguère, mature hardwood forest, Lindgren funnel traps (4, AFC); Meduxnekeag Valley Nature Preserve, 46.1900°N, 67.6700°W, 7.VI.2007, R. P. Webster, hardwood forest, under bark of standing dead beech (1, RWC). **Charlotte Co.**, 10 km NW of New River Beach, 45.2110°N, 66.6170°W, 15-29.VI.2010, R. Webster & C. MacKay, old growth eastern white cedar forest, Lindgren funnel trap (1, RWC). **Northumberland Co.**, 12.0 km SSE of Upper Napan near Goodfellow Brook, 46.8943°N, 65.3810°W, 23.V.2007, R. P. Webster, recent clearcut, under bark of spruce log (1, RWC). **Queens Co.**, Grand Lake near Scotchtown, 46.8762°N, 66.1816°W, 30.IV.2006, R. P. Webster, oak forest, under bark of oak (1, RWC); same locality data, collector, and forest type, 19.IX.2006, in decayed log covered with gilled mushrooms and polypore fungi (1, RWC); Cranberry Lake P.N.A, 46.1125°N, 65.6075°W, 24.IV-5.V.2009, 12-21.V.2009, R. Webster & M.-A. Giguère, old red oak forest, Lindgren funnel traps (5, AFC); Grand Lake Meadows P.N.A., 45.8227°N, 66.1209°W, 4-19.V.2010, 19-31.V.2010, R. Webster, C. MacKay, M. Laity, & R. Johns, old silver maple forest with green ash and seasonally flooded marsh, Lindgren funnel traps (2, AFC). **Restigouche, Co.**, Dionne Brook P.N.A., 47.9030°N, 68.3503°W, 31.V-15.VI.2011, M. Roy & V. Webster, old-growth northern hardwood forest, Lindgren funnel traps (3, AFC, NBM); same locality and collectors but 30.V-15.VI.2011, old-growth white spruce and balsam fir forest, Lindgren funnel traps (4, AFC, NBM). **Sunbury Co.**, Acadia Research Forest, 45.9866°N, 66.3841°W, 13-19.V.2009, R. Webster & M.-A. Giguère, mature (110-year-old) red spruce forest with scattered red maple and balsam fir, Lindgren funnel traps (3, AFC). **York Co.**, Charters Settlement, 45.8340°N, 66.7450°W, 20.V.2007, R. P. Webster, mature mixed forest, in polypore fungi on *Populus* log (1, RWC); Canterbury near Browns Mtn. Fen, 45.8876°N, 67.6560°W, 3.VIII.2006, R. P. Webster, hardwood forest, on slightly dried *Pleurotus* sp. on sugar maple (1, RWC); 15 km W of Tracy off Rt. 645, 45.6848°N, 66.8821°W, 25.IV-4.V.2009, 25.V-1.VI.2009, 1-8.VI.2009, 15-21.VI.2009, R. Webster & M.-A. Giguère, old red pine forest, Lindgren funnel traps (4, AFC); Charters Settlement, 45.8395°N, 66.7391°W, 1-5.VI.2011, R. P. Webster, mixed forest, flight intercept trap (1, NBM).

**Map 6.**
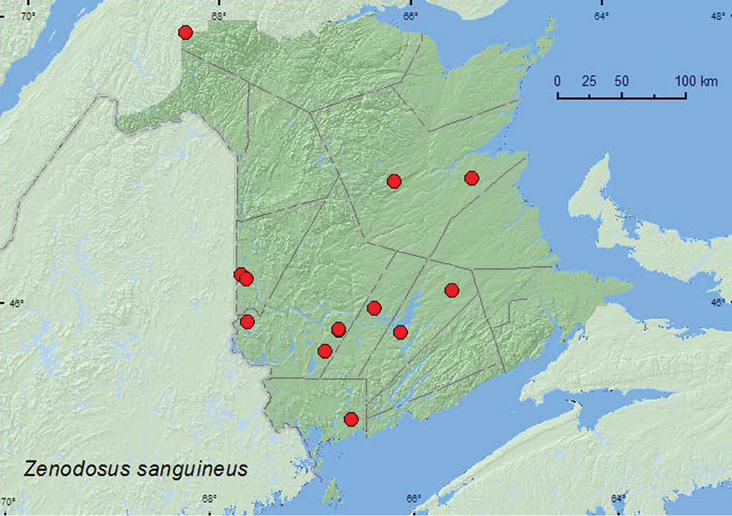
Collection localities in New Brunswick, Canada of *Zenodosus sanguineus*.

##### Collection and habitat data.

In New Brunswick, this species was captured in Lindgren funnel traps deployed in various deciduous and coniferous forest types. These included a mature hardwood forest, an old red oak forest, an old silver maple (*Acer saccharinum* L.) forest, an old-growth northern hardwood forest, a mature mixed forest, an old red pine forest, a mature red spruce forest, an old-growth eastern white cedar forest, and an old-growth white spruce (*Picea glauca* (Moench) Voss) and balsam fir (*Abies balsamea* (L.) Mill.) forest. Adults with micro-habitat data were collected from decaying fleshy polypore fungi and a partially dried polypore fungus on dead, standing trees, in a decayed log covered with gilled mushrooms and polypore fungi, on a slightly dried *Pleurotus* sp. on a sugar maple, in a polypore fungi on a *Populu*s log, under bark of a dead, standing American beech tree, and under bark of a spruce and a red oak log. Adults were collected during April, May, June, August, and September.

##### Distribution in Canada and Alaska.

ON, QC, NB, NS, PE (McNamara 1991; Majka 2006). Majka (2006) reported this species for the first time from New Brunswick on the basis of one specimen from Fredericton (York Co.) collected in 1987. This species is common and widespread in the province based on these collections.

### Subfamily Tillinae Fischer von Waldheim, 1813

#### Cymatodera bicolor

(Say, 1825)

http://species-id.net/wiki/Cymatodera_bicolor

Map 7

##### Material examined.

**New Brunswick, Carleton Co.**, Meduxnekeag Valley Nature Preserve, 46.1957°N, 67.6803°W, 10.VI.2005, R. P. Webster, mixed forest, u.v. light trap (1, RWC). **Queens Co.**, Cranberry Lake P.N.A, 46.1125°N, 65.6075°W, 29.VI-7.VII.2011, 20.VII-4.VIII.2011, M. Roy & V. Webster, old red oak forest, Lindgren funnel traps in forest canopy (8, AFC, NBM, RWC); Grand Lake Meadows P.N.A., 45.8227°N, 66.1209°W, 21.VI-5.VII.2011, M Roy & V. Webster, old silver maple forest and seasonally flooded marsh, Lindgren funnel trap in forest canopy (1, RWC).

**Map 7.**
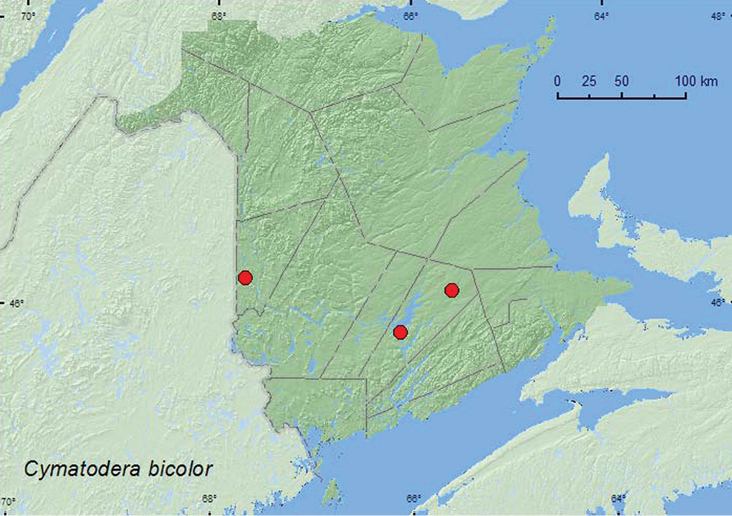
Collection localities in New Brunswick, Canada of *Cymatodera bicolor*.

##### Collection and habitat data.

One specimen from New Brunswick was captured in an ultraviolet light trap in a mixed forest area. Others were captured in Lindgren funnel traps deployed in the canopy of red oaks in an old red oak forest and in the canopy of a silver maple in a silver maple forest. Adults were captured during June, July, and August.

##### Distribution in Canada and Alaska.

ON, QC, **NB**, NS (McNamara 1991). Only one previous specimen of this species was known from the Maritime provinces (Kings Co., Kentville, specimen in CNC) (Majka 2006). Majka (2006) considered this specimen was either from an isolated population in Annapolis Valley of Nova Scotia or a wind-blown stray.

### Subfamily Clerinae Latreille, 1802

#### Thanasimus formicarius

Linnaeus, 1758**

http://species-id.net/wiki/Thanasimus_formicarius

Map 8

##### Material examined.

**Nova Scotia, Halifax Co.**, (Halifax) Point Pleasant Park, 44.6226°N, 63.5689°W, 11.VII.2001, 8.VIII.2001, J. Sweeney, Lindgren funnel traps, tree blend lure (2, AFC, CNC). **Quebec,** Berthierville, late 1940’s, Frère Adrien Robert (UMC).

**Map 8.**
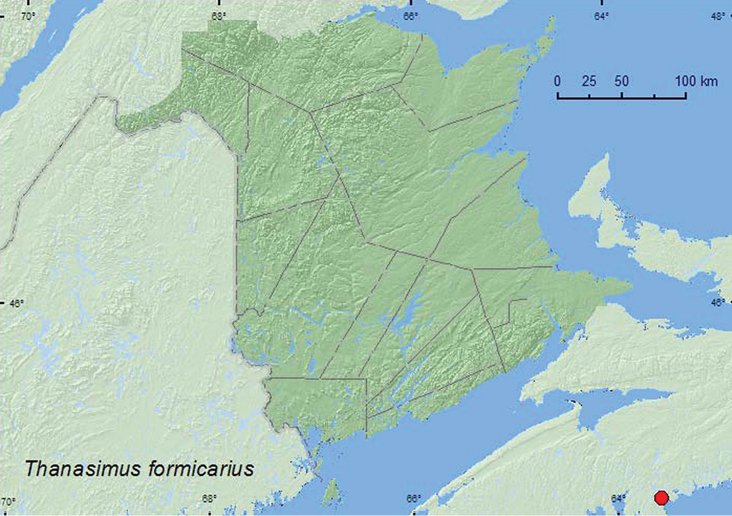
Collection localities in Nova Scotia, Canada of *Thanasimus formicarius*.

##### Collection and habitat data.

In Europe, *Thanasimus formicarius* is a well-known predator of bark beetles (Weslien and Regnander 1992). Two adults from Nova Scotia were captured in Lindgren funnel traps baited with tree blend lure (spruce volatiles) and EtOH deployed in a red spruce stand. Adults were collected during July and August.

##### Distribution in Canada and Alaska.

QC, **NS**. This old-world species was introduced into North America to control the bark beetle, *Dendroctonus frontalis* Zimmermann in 1892 and in the late 1900s (Opitz 2002). It is not known if this species is established in Nova Scotia or if these specimens represents an interception of individuals that may have emerged from softwood packing material used as dunnage in shipping containers arriving in the port of Halifax from Europe. No additional specimens have been collected at or near this site despite extensive trapping from 2001–2011 in the Halifax–Dartmouth area with similarly baited funnel traps or black-panel intercept traps (Alpha Scents, Portland, OR). There is also a specimen in the Ouellet-Robert Collection (Université de Montréal) from Berthierville, Quebec, collected by A. Robert in the 1940s during his studies on Dutch elm disease (Serge Laplante, personal communication). There have been no additional specimens reported from Quebec.

### Family Melyridae Leach, 1815

Mayor (2002) presented an overview of the Melyridae (the soft-winged flower beetles) of North America. Adult Melyridae feed on both plant and animal matter, such as small arthropods, and especially on pollen and nectar. Larvae are predators and scavengers, and feed on detritus, fungi, and small arthropods, including their larvae and eggs (Mayor 2002). In Canada, most species occur only in the West and only the introduced *Malachius aeneus* (Linnaeus) was recorded from Maritime provinces and New Brunswick (Bright 1991). Majka (2005) reviewed the Melyridae of the Maritime provinces and newly reported *Collops vittatus* (Say) and *Nodopus flavilabris* (Say) (as *Anthocomus flavilabris* (Say)) from New Brunswick. Here, we report *Attalus morulus* (LeConte) and *Dolichosoma foveicolle* (Kirby) for the first time for New Brunswick and the Maritime provinces (Table 1).

### Subfamily Malachiinae Fleming, 1821

#### Attalus morulus

(LeConte, 1852)**

http://species-id.net/wiki/Attalus_morulus

Map 9

##### Material examined.

**New Brunswick, Northumberland Co.**, Blueberry Rd., off Hwy 8, 47.3210°N, 65.4229°W, 24.VII.2005, R. P. Webster, jack pine forest, on foliage of jack pine (1, RWC). **York Co.**, 15 km W of Tracy off Rt. 645, 45.6848°N, 66.8821°W, 29.VII–4.VIII.2009, R. Webster & M.-A. Giguère, old red pine forest, Lindgren funnel trap (1, RWC); same locality data and forest type, 13–27.VII.2010, R. Webster & C. MacKay, Lindgren funnel traps (2, AFC, RWC).

**Map 9.**
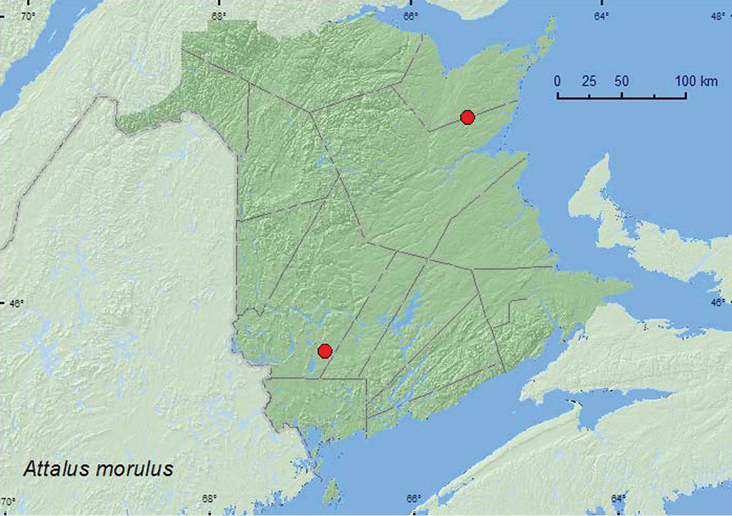
Collection localities in New Brunswick, Canada of *Attalus morulus*.

##### Collection and habitat data.

*Attalus morulus* were captured in Lindgren funnel traps deployed in an old-growth red pine forest. One individual was collected from foliage of jack pine (*Pinus banksiana* Lamb.) in a jack pine forest. Adults were captured during July and August.

##### Distribution in Canada and Alaska.

BC, QC, **NB** (Bright 1991). *Attalus morulus* was previously reported from Connecticut and New York, in northeastern USA (Downie and Arnett 1996).

### Subfamily Dasytinae Laporte, 1840

#### Dolichosoma foveicolle

(Kirby, 1837)**

http://species-id.net/wikiDolichosoma_foveicolle

Map 10

##### Material examined.

**New Brunswick, Albert Co.**, Shepody N.W.A., Mary’s Point Section, 45.7320°N, 64.6765°W, 16.VI.2004, R. P. Webster, margin of salt marsh near forest, sweeping (1, RWC). **Gloucester Co.**, near Acadian Historical Village, 47.7873°N, 65.0756°W, 29.VI.2006, R. P. Webster, inland margin of salt marsh, sweeping vegetation (6, NBM, RWC). **Sunbury Co.**, Burton, near Sunpoke Lake, 45.7662°N, 66.5526°W, 20.VI.2007, R. P. Webster, seasonally flooded marsh, sweeping vegetation (1, RWC). **York Co.**, Mazerolle Settlement, 45.8765°N, 66.8260°W, 8.VI.2008, R. P. Webster, beaver meadow, sweeping vegetation along brook margin (2, RWC).

**Map 10.**
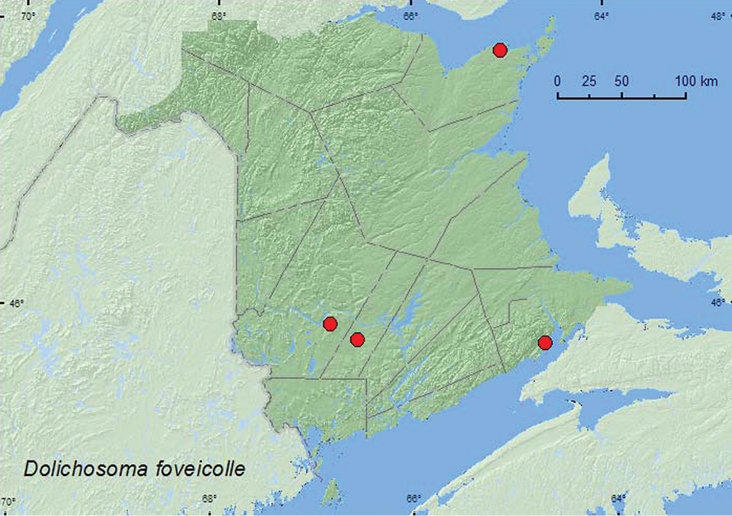
Collection localities in New Brunswick, Canada of *Dolichosoma foveicolle*

##### Collection and habitat data.

Adults were found in June on the inner margin of salt marshes, seasonally flooded (freshwater) marshes, and in a beaver meadow, and were collected by sweeping the marsh vegetation.

##### Distribution in Canada and Alaska.

BC, AB, MB, ON, QC, **NB**, (Bright 1991).

## Supplementary Material

XML Treatment for Grynocharis quadrilineata

XML Treatment for Ostoma fraterna

XML Treatment for Thymalus marginicollis

XML Treatment for Calitys scabra

XML Treatment for Tenebroides corticalis

XML Treatment for Zenodosus sanguineus

XML Treatment for Cymatodera bicolor

XML Treatment for Thanasimus formicarius

XML Treatment for Attalus morulus

XML Treatment for Dolichosoma foveicolle
